# Comparisons of optically monitored small-scale stirred tank vessels to optically controlled disposable bag bioreactors

**DOI:** 10.1186/1475-2859-8-44

**Published:** 2009-08-05

**Authors:** Michael A Hanson, Kurt A Brorson, Antonio R Moreira, Govind Rao

**Affiliations:** 1 Center for Advanced Sensor Technology, Chemical and Biochemical Engineering Department, University of Maryland Baltimore County, Baltimore, MD, 21250, USA; 2 Division of Monoclonal Antibodies, Center for Drug Evaluation and Research, Food and Drug Administration, 10903 New Hampshire Ave., Silver Spring, MD 20903, USA; 3 Wyeth Pharmaceuticals, Manufacturing Science and Technology, 401 N. Middletown Rd., Pearl River, NY 10965, USA

## Abstract

**Background:**

Upstream bioprocesses are extremely complex since living organisms are used to generate active pharmaceutical ingredients (APIs). Cells in culture behave uniquely in response to their environment, thus culture conditions must be precisely defined and controlled in order for productivity and product quality to be reproducible. Thus, development culturing platforms are needed where many experiments can be carried out at once and pertinent scale-up information can be obtained.

**Results:**

Here we have tested a High Throughput Bioreactor (HTBR) as a scale-down model for a lab-scale wave-type bioreactor (CultiBag). Mass transfer was characterized in both systems and scaling based on volumetric oxygen mass transfer coefficient (k_L_a) was sufficient to give similar DO trends. HTBR and CultiBag cell growth and mAb production were highly comparable in the first experiment where DO and pH were allowed to vary freely. In the second experiment, growth and mAb production rates were lower in the HTBR as compared to the CultiBag, where pH was controlled. The differences in magnitude were not considered significant for biological systems.

**Conclusion:**

Similar oxygen delivery rates were achieved in both systems, leading to comparable culture performance (growth and mAb production) across scales and mode of mixing. HTBR model was most fitting when neither system was pH-controlled, providing an information-rich alternative to typically non-monitored mL-scale platforms.

## Background

The upstream stages of a typical bioprocess are, arguably, the most complicated since living organisms are used. Cells in culture behave uniquely in response to their environment, thus culture conditions must be precisely defined and controlled in order for productivity and product quality to be reproducible. Consequently, upstream bioprocess development requires many experiments to optimize media and growth conditions, as well as select the actual production organism.

Development experiments would be most informative if they could be carried out using the same equipment (i.e., the same scale) eventually to be used for the commercial process. However, due to economic and time constraints, strain selection and media and bioreactor development have typically been carried out in low volume, high throughput platforms, such as flasks on the scale of 50 – 6,000 ml and lab-scale bioreactors [1]. The primary concern with development in small-scale platforms is whether cellular behavior exhibited is comparable to what will be observed at commercial-scale. Without monitoring and control of critical parameters pH and dissolved oxygen (DO) in flasks, these parameters could drift to unfavorable levels, thus confounding cell viability, strain productivity or media component dependence [2-4]. Lab-scale bioreactors can monitor and control DO and pH but they are comparatively low throughput (i.e., they run one relatively large culture at a time).

Recently we described an ml-scale high throughput bioreactor system (HTBR) which incorporates advances over the above described systems [5]. The parallel reactor design allows up to twelve cultures to be carried out simultaneously. Each individual 35 ml (working volume) bioreactor contains a two-paddle impeller for stirring, gas in- and out-lets for aeration, and optical sensors for measurement of pH and DO. We have also shown the optical sensors function equivalently as traditionally electrochemical probes for short-term culturing [6].

Use of wave-type bioreactors in industrial settings has increased steadily since their conception in the mid-1990s. These reactors were developed by Singh and coworkers, then at Schering Plough, where they characterized mass transfer and cultivated CHO, NS0, HEK 293, and Sf9 cells under a variety of conditions [7]. The technology was further developed by the inventors as the primary product of Wave Biotech, now a subsidiary of GE Corporation . Disposable bioreactors are very attractive for industrial settings because they are single-use and, thus, do not require cleaning and sterilization nor the validation of these steps. Disposable bag systems with optical sensors for both pH and DO (CultiBags) by Sartorius-Stedim  have been developed to allow both monitoring and control of these two critical cell culture parameters.

In the work presented here, our initial HTBR publication [5] is expanded upon by testing whether the system is a good scale-down model for wave-type bioreactors. Oxygen mass transfer characterization and cell culture experiments were carried out to compare the two systems.

## Results and Discussion

### Mass Transfer Characterization

The first step in establishing a scale-down model was to establish environments at both scales where oxygen delivery rate was approximately equal. To do this, volumetric oxygen mass transfer coefficient (*k*_*L*_*a*) was characterized in both systems with respect to agitation parameters and air flow rate. When determining *k*_*L*_*a *in the CultiBag and HTBR, the oxygen transfer rate into the optical sensing foils had to be taken into consideration and the transfer constants were determined to be 47.4 and 10.3 h^-1^, respectively. Transfer rate is a function of the sensor material, its thickness, and the boundary layers on either side of the material. Also, embedded in the transfer constant value is sensing reaction rate, which is based on the sensing chemistry. Although both reactor systems use fluorescence lifetime sensing for oxygen, the optical sensors are from different vendors, PreSens  and Fluorometrix , for CultiBag and HTBR, respectively, and the sensing chemistries are different. Detailed information about sensing chemistries and sensing foil construction is proprietary. All of these factors could have contributed to the 4.6-fold difference in transfer coefficients. Ultimately, the transfer rates were characterized to insure equal mass transfer in the experiments. The objective of the paper is to show comparable cell culture performance; in-depth sensing system analyses are beyond the scope.

**Figure 1:**
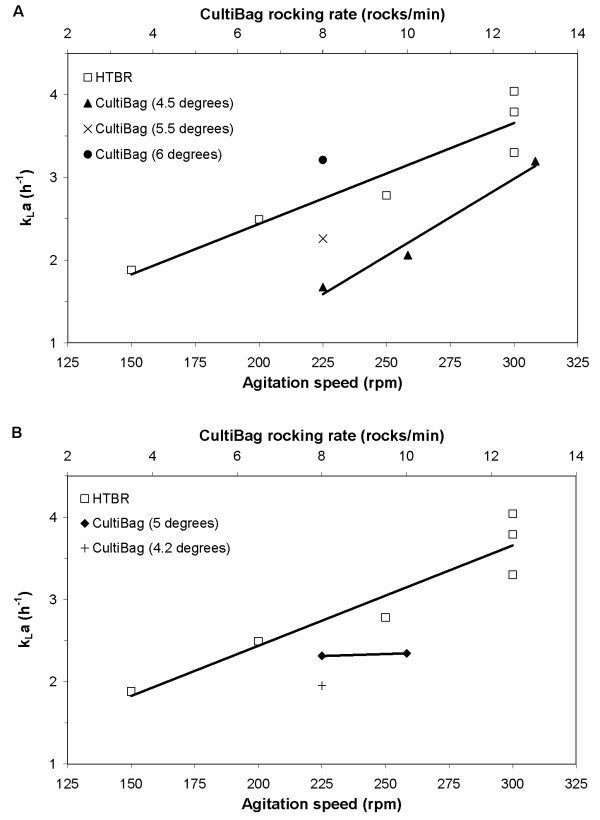
***k_*L*_a *in HTBR and CultiBag**. *k*_*L*_*a *in HTBR and CultiBag at various agitation speeds and rocking rates and angles. (A) HTBR – 35 mL, CultiBag – 2 L. (B) HTBR – 35 mL, CultiBag – 3.5 L.

Figure 1 shows how *k*_*L*_*a *changes with impeller rotational rate in the HTBR, and rocking rate and angle in the CultiBag, where both systems were aerated through the headspace only. *k*_*L*_*a *increased linearly with impeller rotational rate in the HTBR and rocking rate in the CultiBag (2 L, Figure 1A). At 3.5 L (Figure 1B), *k*_*L*_*a *in the CultiBag was unresponsive to a small increase in rocking rate from 8 to 10 rocks/min. The same HTBR data is used in both the 2 and 3.5 L plots, as the liquid volume was always 35 ml.

The measured CultiBag k_L_a (2 – 4 h^-1^) were similar to those observed in other wave-type bioreactor characterization studies at comparable agitation and air flow rates [7,8]. Characterization conditions in the HTBR were chosen to give mass transfer coefficients in the same approximate range. This range is less than what has been achieved in other stirred small-scale bioreactor systems where *k*_*L*_*a *of 360 h^-1 ^[9] and 1,500 h^-1 ^[10] have been observed. However, our system volume was 4-fold larger, and for the purpose of the study, low rates of impeller agitation and aeration through the headspace was chosen.

### Cell Culture Comparisons

After showing the systems could provide similar environments in terms of oxygen delivery, culture performance experiments focusing on cell growth and mAb production were carried out. The first experiment, Bioreactor Comparison 1, was conducted without controlling pH nor DO in the CultiBag, enabling a direct comparison to the HTBR, which is not capable of controlling either parameter. A working volume of 2 L in the CultiBag resulted in approximately a 60× scale-down to the 35 ml HTBR. Rocking rate and angle in the CultiBag and agitation rate in the HTBR were manipulated to achieve k_L_a of 2.06 h^-1^and 2.49 h^-1^, respectively.

**Figure 2:**
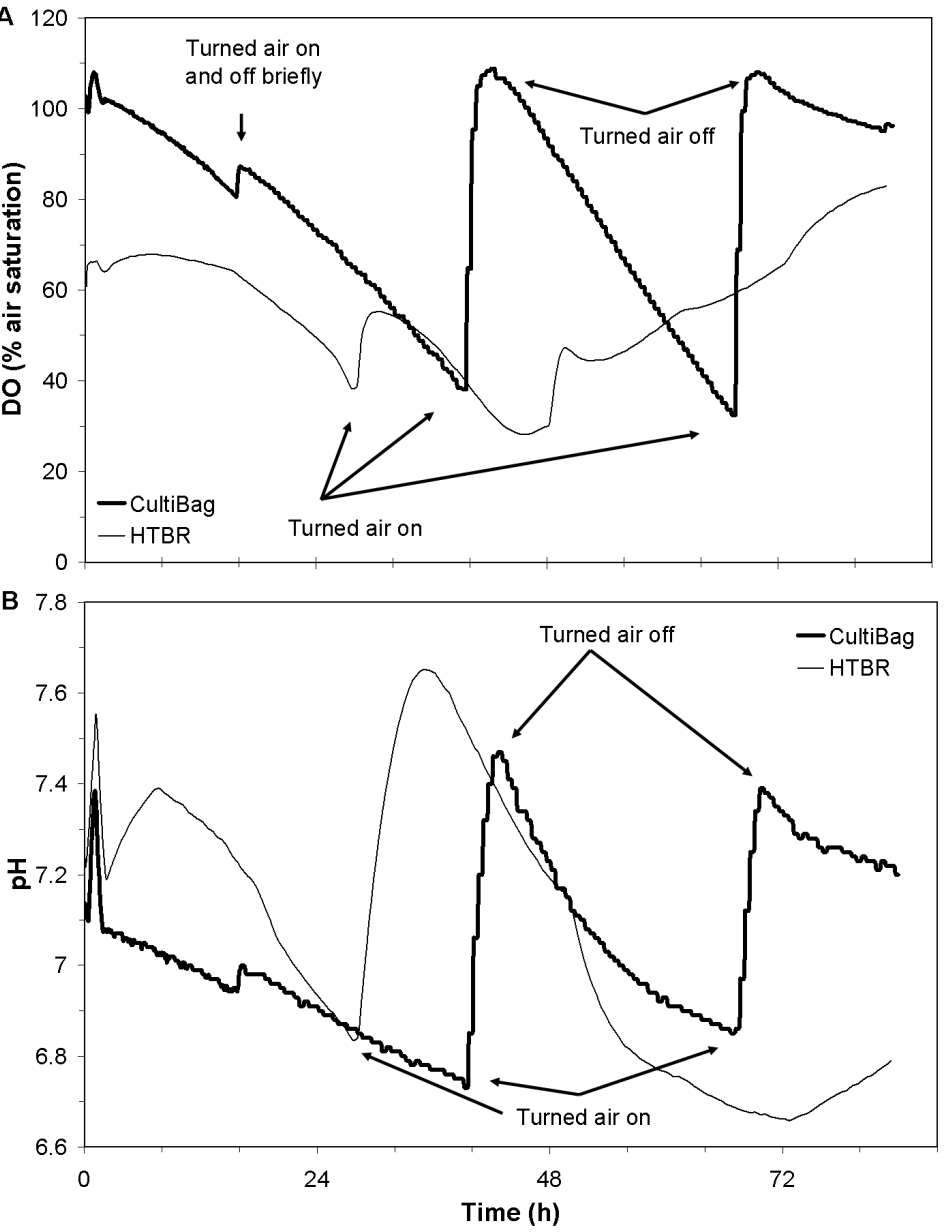
**DO and pH profiles: Bioreactor Comparison 1**. DO (A) and pH (B) profiles during Bioreactor Comparison 1.

Approximately equalizing mass transfer in the two systems resulted in an accurate scale-down as demonstrated by the similar negative DO slopes with respect to time from 16 – 28 and 30 – 40 hours in Figure 2A, implying similar oxygen delivery rates to the cells by the reactors, given approximately equal oxygen uptake rates (OURs). Upon turning on air in both reactors, the CultiBag recovered completely to saturation, but the HTBR only responded with a slight increase, eventually continuing to decrease 1 – 2 h later. This was unexpected, considering that the HTBR *k*_*L*_*a *was slightly higher than the CultiBag's (2.49 to 2.06 h^-1^). It is possible the increased ability to deliver oxygen in the CultiBag was a result of bag over-pressurization due to a partially blinded exhaust filter, an issue that has been observed by others [8]. Although a filter heater is provided which aims to evaporate any condensed liquid in the exhaust filter, splashing from the wave motion of the cell broth could lead to filter wetting and blinding. In this experiment, liquid present in the filter housing and an increase in bag pressure both point to filter blinding. Although the CultiBag system proved more controllable from a DO standpoint by simply turning air on and off, it should be noted that the DO concentration in the HTBR never dropped below an unacceptable level (i.e., < 20%).

From a qualitative perspective, the culture pH profiles from the two systems, shown in Figure 2B, further support successful scale-down. The pH progressed in opposite directions in the two systems for approximately the first 8 h. The small headspace volume relative to culture volume in the HTBR enabled rapid replacement of off-gases by air, thus facilitating the stripping of dissolved CO_2 _by air in the culture media, and increasing the pH. Following this initial difference, pH in both reactor systems fluctuated, presumably, due to acidic byproduct secretion and stripping. The former phenomenon overcame the latter in the CultiBag once air was turned off, both times. pH naturally dropped in the HTBR even though the air stayed on, as was observed in other HTBR experiments [5]. It should be noted that the pH in neither system went outside of a range compatible with culture viability (i.e. 6.6 – 7.6).

The DO and pH profiles demonstrated comparable behaviour across scales and reactor type, but the most important evidence of successful scale-down was equivalent culture growth and mAb production. The CultiBag and HTBR cell densities both peaked at 60 h into the experiment at 1.28 and 1.18 ± 0.24 × 10^6 ^cells/ml, respectively, as shown in Table 1. IgG_3 _was produced at 37 and 44 ± 12 pg/cell-day in the CultiBag and HTBR, respectively. Similar growth and specific productivities would thus ensure comparable reactor volumetric productivity upon scale-up.

**Table 1:** Cell culture performance parameters

	Bioreactor Comparison 1		Bioreactor Comparison 2	
Parameter	CultiBag	HTBR	CultiBag	HTBR

culture volume	2 L	35 ml	3.5 L	35 ml
peak vcd (× 10^6 ^cells/ml)	1.28	1.18 ± 0.24	0.76	0.70 ± 0.18
final IVCD (× 10^6 ^cells/ml-h)	59.70	50.63 ± 9.89	38.39	23.85 ± 8.05
peak mAb concentration (mg/l)	92.92	92.16 ± 7.08	42.41	26.65 ± 4.51
average growth rate (1/h)	0.026	0.029 ± 0.007	0.036	0.022 ± 0.005
average mAb productivity (pg/cell-d)	37.35	44.87 ± 12.08	32	28.64 ± 5.70

Summary of growth and mAb production during the bioreactor comparisons. All reactors from the same comparison inoculated from the same pool of cells. N = 4 and 3 for HTBR bioreactors in Bioreactor Comparisons 1 and 2, respectively.

**Figure 3:**
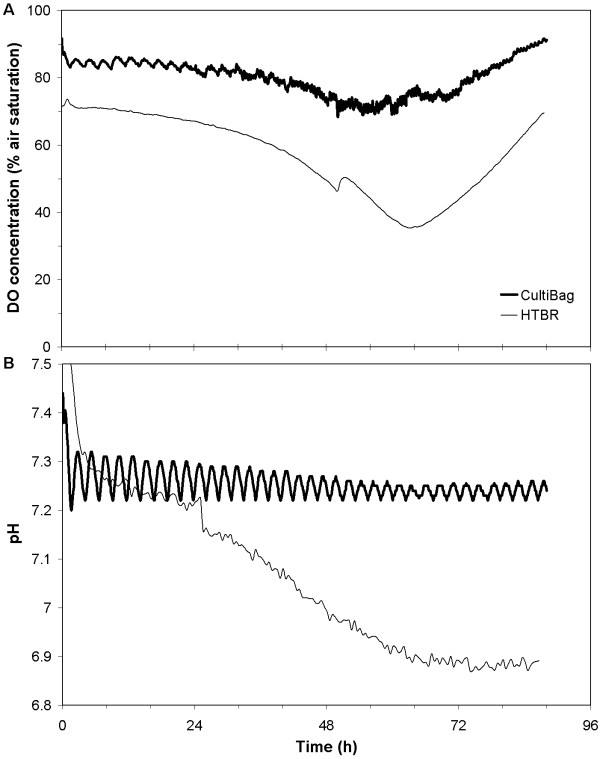
**DO and pH profiles: Bioreactor Comparison 2**. DO (A) and pH (B) profiles during Bioreactor Comparison 2.

In a second cell culture comparison experiment, the culture volume of the CultiBag was 3.5 L, resulting in a 100-fold scale-down to the HTBR. The CultiBag and HTBR were operated using the same agitation parameters as were used in Bioreactor Comparison 1, resulting in *k*_*L*_*a *of 2.34 and 2.49 h^-1^, as shown in Figure 1B. These oxygen mass transfer coefficients and the observed cellular behaviours led to the DO profiles shown in Figure 3A, which were similar for both systems. Oscillations in CultiBag DO profiles are a result of intermittent CO_2 _gas addition for pH control, which strips DO from the media. DO started lower in the HTBR in comparison to the CultiBag due to the time delay in inoculation, as was the case in the first experiment. The overall similarity in DO profiles among both systems is strong evidence that the HTBR is an appropriate scale-down model when scaled on the basis on volumetric oxygen mass transfer coefficient (*k*_*L*_*a*).

In Bioreactor Comparison 2, pH was controlled in the CultiBag by way of intermittent CO_2 _injections into the headspace air stream. The pH profiles in Figure 3B show oscillations around the pH set point of 7.3 for the CultiBag. HTBR pH was maintained in the range of 6.9 – 7.3 for the vast majority of the experiment by aeration with 5% CO_2 _in air. This range of 0.4 pH units is relatively tight in comparison the 6.7 – 7.7 pH range observed in Bioreactor Comparison 1. Thus simply aerating with a CO_2_:air mixture improves the HTBR's ability to behave similarly to a pH-controlled reactor. The pH profile in the CultiBag demonstrates the ability to control pH in wave-type systems using optical sensors for monitoring. Anecdotally, pH control has been a problem in wave-type systems when using electrochemical pH probes.

Culture performance was less similar in Bioreactor Comparison 2. In terms of all growth and mAb production parameters (peak viable cell density, peak mAb concentration, integrated viable cell density, average growth rate, and specific productivity), the CultiBag out-performed the HTBR (Table 1). However, CultiBag peak cell density and specific productivity were less than one standard deviation from the HTBR mean. And for the other three parameters, the HTBR value was < 40% lower than the CultiBag value, a difference which was not considered significant for biological systems. One explanation for increased performance over the HTBR may have been the ability to tightly control pH in the CultiBag. Differences in pH have been shown to affect specific productivity by others [11].

Systems traditionally used for mL-scale development (e.g., flasks) also do not have pH control. Thus, the HTBR is similar in that regard. What makes the HTBR an improvement over existing systems is that pH and DO can be monitored, allowing cause and effect relationships be drawn between these parameters and culture performance. Use of pH control in Comparison 2 serves several purposes. First, it demonstrates control precision capable in wave-type systems with optical pH sensors. Second, it demonstrates the need for pH control at mL-scale; an improvement that has been implemented in the next generation HTBR.

## Conclusion

Two bioreactor systems spanning mode of mixing and scale were used to culture IgG_3 _producing hybridoma cells. Mass transfer was characterized in both systems and scaling based on volumetric oxygen mass transfer coefficient (*k*_*L*_*a*) was sufficient to give similar DO trends. HTBR and CultiBag cell growth and mAb production rates were approximately equal in the first experiment where DO and pH were allowed to vary freely. In the second experiment, growth and mAb production levels were higher in the CultiBag, where pH was more tightly controlled. In closing, since similar oxygen delivery rates could be achieved, and culture performance (growth and mAb production) was consistent across scales, the HTBR shows promise as a high-throughput scale-down model for wave-type bioreactors.

## Methods

### Bioreactor Descriptions

Cells were grown in two bioreactor systems: a CultiBag RM Optical (Sartorius-Stedim) and an HTBR (Fluorometrix, Stowe, MA). For the CultiBag cultures, 10 L polyethylene bags were used, each containing optical sensor foils for pH and DO. The HTBR is capable of simultaneously carrying out 12 cultures where each reactor vessel has a maximum 35 ml working volume and optical sensors for DO and pH [5]. The HTBR used for this study is only capable of monitoring, not controlling DO and pH.

### *k*_*L*_*a *and Optical DO Sensor Patch Responses Measurements

*k*_*L*_*a *was determined at different agitation speeds in the HTBR and, volume, rocking angles and rates in the CultiBag using the dynamic "gas out-gas in" method [12]. When the sensor response time is taken into consideration to account for the time required for DO diffusion from the liquid bulk into the sensor foils [13], the oxygen mass balance can be solved explicitly for the DO concentration in the sensor patch, *C*_*P*_:

(1)

where  is the DO concentration at the air-liquid interface, *C*_0 _is the initial DO concentration in the bulk liquid, and *k*_*P *_is the volumetric mass transfer coefficient into the sensor foil, which was determined by independently by modelling the response in *C*_*P *_to an ideal step change in liquid bulk DO. *k*_*L*_*a *can be determined upon fitting *C*_*P*_-time profiles to Equation 1.

### Cell Line and Growth Media

The cell line is a Sp2/0 myeloma/mouse hybridoma cell line (2055.5); it produces an IgG_3 _mAb specific for *Neisseria meningitidis *capsular polysaccharides (anti-MCPS) [14]. Cells were grown in CD Hybridoma (Gibco, Carlsbad, CA), a protein-free media supplemented to 2 mM glutamine (HyClone, Laboratories Inc., Logan, UT), 100 units/ml penicillin (HyClone), 100 μg/ml streptomycin (HyClone), 1 g/l PF-68 (MP Biomedicals, LLC, Aurora, OH) and 3.5 × 10^-4^% β-mercaptoethanol (v/v) (Sigma, St. Louis, MO). Working cultures were maintained in spinner flasks housed in a water-jacketed incubator (Napco, Winchester, VA) at 37°C and 50% relative humidity with a headspace CO_2 _composition of 5% in air.

### Cell Culture Experiments

Two separate bioreactor comparison experiments were carried out (Bioreactor Comparisons 1 and 2). Within each experiment, all bioreactors used were inoculated from the same pool of cells. Cells were counted using a hemocytometer where viability was assessed using the Trypan Blue exclusion method. IgG_3 _product was measured using a sandwich-type ELISA [15].

In the first experiment, Bioreactor Comparison 1, the HTBR and CultiBag were used at 35 ml and 2 L, respectively. Neither pH nor DO concentration was controlled in either system. Cultures were aerated via the headspace with air at 0.3 VVM in the HTBR and 0.05 VVM in the CultiBag when DO concentration dropped below 40% air saturation. Agitation speed in the HTBR (200 rpm) and rocking rate (10 rocks/min) and angle (4.5°) in the CultiBag were chosen to approximately match *k*_*L*_*a *in the two systems.

In the second experiment, Bioreactor Comparison 2, the HTBR was again used at 35 ml, however the volume of the CultiBag was 3.5 L, resulting in a 100× scale-up relative to HTBR. pH was controlled in the CultiBag via intermittent CO_2 _addition. When not introducing CO_2_, there was constant headspace aeration of air at 0.05 VVM. In the HTBR, a 5% CO_2 _in air solution was continuously headspace aerated at 0.3 VVM. DO was not controlled in either system. *k*_*L*_*a *was again approximately equalized in the two systems. The agitation speed of the HTBR was 200 rpm. The CultiBag was rocked at a rate of 10 rocks/min at an angle of 4.5°.

## Competing interests

The HTBR technology described in this paper has been licensed to Fluorometrix, in which G. Rao has an equity position. A sub-license has been issued by Fluorometrix to Sartorius-Stedim Biotech. Views expressed in this article are those of the authors and not necessarily of the US FDA or the US government. Discussion of the individual cell culture devices does not constitute endorsement by the US FDA or the US government.

## Authors' contributions

MAH carried out the experiments, wrote the text, and was the primary data analyzer. KAB, ARM, and GR assisted with experimental design, data analysis, and were the text editors. All authors read and approved the final manuscript.
